# Laminated Veneers with Stratified Feldspathic Ceramics

**DOI:** 10.1155/2018/5368939

**Published:** 2018-12-06

**Authors:** Henrique Tuzzolo Neto, Wagner Ferreira do Nascimento, Larissa Erly, Rodrigo Alves Ribeiro, Jorge de Sá Barbosa, Jessica Mina Zambrana, Lariça Barbosa Raimundo, Cristiane da Silva Mendes, Ivan Pereira da Silva, Alfredo Mikail Melo Mesquita, Daniela Miranda Richarte de Andrade Salgado, Claudio Costa

**Affiliations:** ^1^Department of Prosthetic Dentistry, School of Dentistry, Metropolitan University of Santos, Santos, Brazil; ^2^Department of Stomatology, School of Dentistry, University of São Paulo, São Paulo, Brazil; ^3^Department of Prosthetic Dentistry, School of Dentistry, University Paulista, São Paulo, Brazil

## Abstract

Currently, there has been a growing demand for restorations of anterior teeth in the routine of doctors in dental offices. With advances in adhesive technology and good properties of the available ceramics, the use of ceramic veneers has been offered as a treatment option for cases where teeth have minor color changes and shape and position changes. Through careful treatment planning, it is possible to conservatively use ceramic veneers in the treatments, applying techniques with minimal wear of the teeth and obtaining excellent aesthetic results with mechanical stability and biocompatibility with the periodontal tissues and clinical longevity. This clinical case report was used to design the digital smile, which guided the production of the diagnostic waxing of the superior model. Silicone guides were then made to create the mock-up, orienting enamel/dentin wear and assisting the preparation of the crowns with bis-acryl resin. When the wear was finished, the gingival separation was performed using the double-thread technique. In the molding process, the second wire was withdrawn and the molding material flowed into the gingival groove, copying the terminal region. After analysis of the quality of the impression, the temporary crowns were made with bis-acryl resin, using the guide. The color of the cementing agent was chosen using a glycerin-based compound which simulates the final color of the cement. After two hours of drying, two different layers of silane were applied for 60 seconds. After the preparation of the piece, gingival isolation and separation were done. The dental substrate was degreased with detergent solution and conditioned with 37% phosphoric acid for 15 seconds and then washed for 45 seconds for subsequent drying, leaving the substrate moistened. The adhesive system was applied for 30 seconds, waiting for the adhesive to penetrate into the conditioned dentinal tubules. A light air jet was applied, and then each tooth was photopolymerized for 30 seconds. The resin cement was placed inside the pieces and placed in position and photopolymerized. The excess cement was removed; after a layer of glycerin in the cervical region, aiming to block the entry of oxygen and polishing was performed. The purpose of this case report was to describe a sequence of clinical steps, from planning to cementation, in a case of aesthetic correction using feldspathic ceramic veneers.

## 1. Introduction

Currently, there has been a growing demand for preventive treatment by patients in dental offices. Such patients are aware of the importance of preventive care and proper oral hygiene. In addition, the interest in aesthetics that can be approached by minimally invasive treatments, as a result of technological, technical, and material advances, is becoming widely known by the population. Ceramic veneers are among the minimally invasive aesthetic treatment options used to improve the tooth shape and color, as well as replacing composite resin restorations.

Ceramic veneers, when well-planned and indicated, provide excellent end result to treatments due to the material property, biocompatibility with the periodontal tissues, and the possibility of being handled in low thicknesses, without harming the resistance or the aesthetics of the material. Thus, procedures are accomplished with minimal wear of the dental tissue, maintaining the color stability [1]. The use of the mock-up technique and bonding procedures are very important to achieve excellent results when working with feldspathic ceramic veneers for the restoration of anterior teeth [2].

The clinical success of feldspathic laminate veneers depends on the appropriate indication and the adequate application of the available materials and techniques, according to the need and the goal of the aesthetic and functional treatment [3–5].

The use of a mock-up prevents excessive or incorrect tooth preparation, as it indicates the exact location and amount of reduction, necessary to obtain the desired tooth color and shape, as well as the confection of the feldspathic ceramic veneers [6].

The purpose of this case report was to describe a sequence of clinical steps, from planning to cementation, in a case of aesthetic correction using feldspathic ceramic veneers.

## 2. Case Report

**Figure 1:**
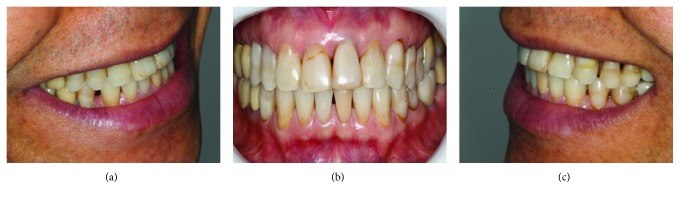
(a) Initial photo of patient's right side. (b) Initial photo frontal view. (c) Initial photo of patient's left side.

**Figure 2:**
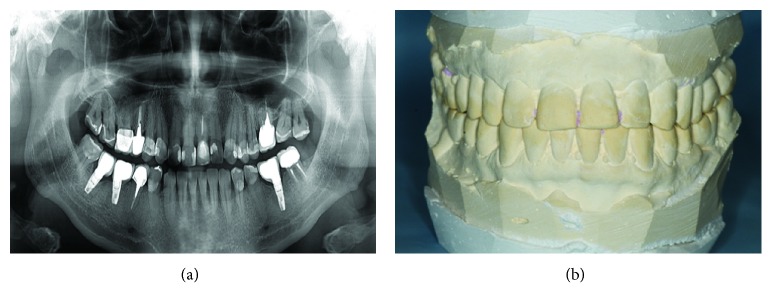
(a) Initial X-rays. (b) Initial study models.

Patient R.C., 52-year-old male subject, sought for dental treatment demonstrating dissatisfaction with the upper anterior teeth aesthetics, which had extensive composite resin restorations, pigmentation, spots, and infiltration (Figure 1). After the anamnesis, as well as the X-rays, photographs and upper and lower arches' evaluation (Figure 2), the case was prepared and an aesthetic rehabilitation treatment was proposed, consisting of a ceramic crown, with zirconia infrastructure in element 15 and laminated veneers, with feldspathic ceramics in elements 11, 12, 13, 14, 21, 22, 23, 24, and 25.

**Figure 3:**
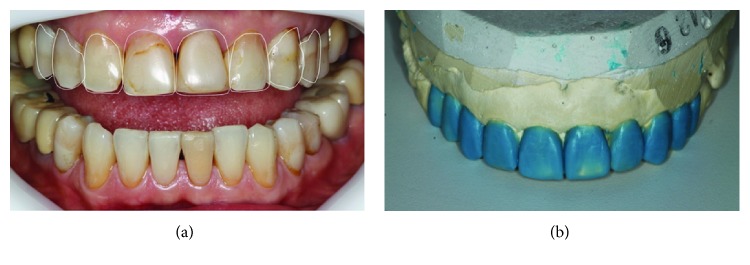
(a) Digital smile design. (b) Wax-up upper arch.

**Figure 4:**
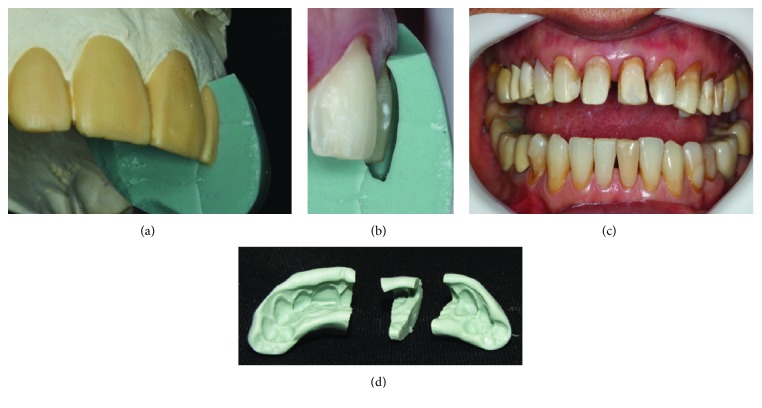
(a) Silicone guide on the wax-up model. (b) Silicone guide cut along the long axis of the tooth. (c) Preparation using the guide. (d) Preparation finalized.

**Figure 5:**
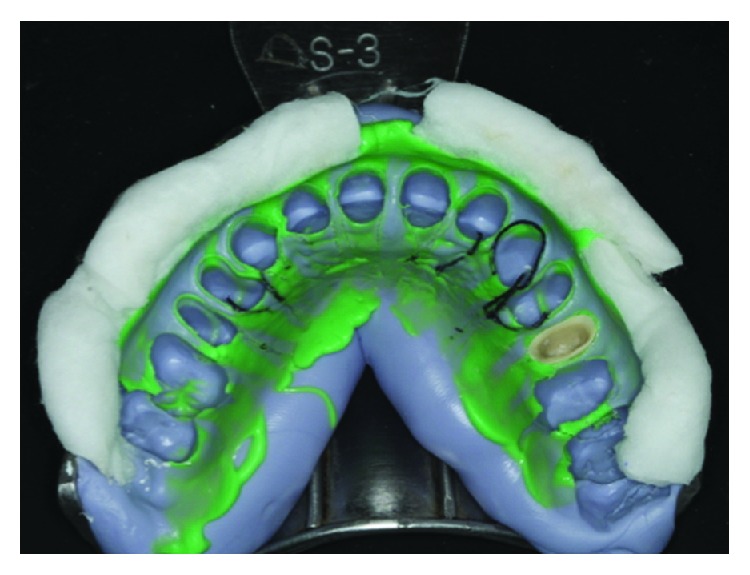
Mold.

**Figure 6:**
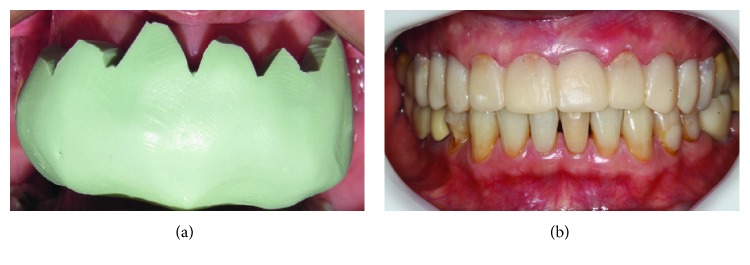
(a) Silicone mold filled with bis-acryl resin. (b) Temporaries in position.

Following the references from the digital smile design, which is performed using pictures of the face and smile of the patient and a computer program, PowerPoint (Microsoft) or Keynote (Mac), it is possible to correct positioning and minor glitches using smile lines and median with teeth proportionality; a diagnostic wax-up of the upper model was produced (Figure 3), and based on it, silicon guides were made to create the mock-up, guide the enamel/dentin preparation, and subsequently assist in the preparation of a temporary crown with bis-acryl resin. Considering tooth 15, a metal-free preparation was made to cover the full crown with zirconia infrastructure; elements 14, 24, and 25 were chosen for inverted-type 4/5 preparations, and for the anterior teeth, tooth preparation for laminated veneers with incisal wear and no overlap was considered for the treatment (Figure 4). When the tooth preparation was finalized, gingival separation was done by applying the double-wire technique. For this, wires no. 000 and no. 00 (Ultrapak, Ultradent), embedded in hemostatic solution (Hemostop, Dentsply), were used. Considering the molding process, at the time of the light A-silicone insertion, the second wire (no. 00) was removed and the material flowed into the gingival sulcus, copying the terminus region. Soon after, the tray with the putty A-silicone was positioned (Flexitime, Heareus Kulzer) (Figure 5). After analyzing the mold quality, the temporary crowns were prepared with A3-colored bis-acryl resin (Structur, Voco), using the silicone guide built on the model with diagnostic wax-up (Figure 6).

**Figure 7:**
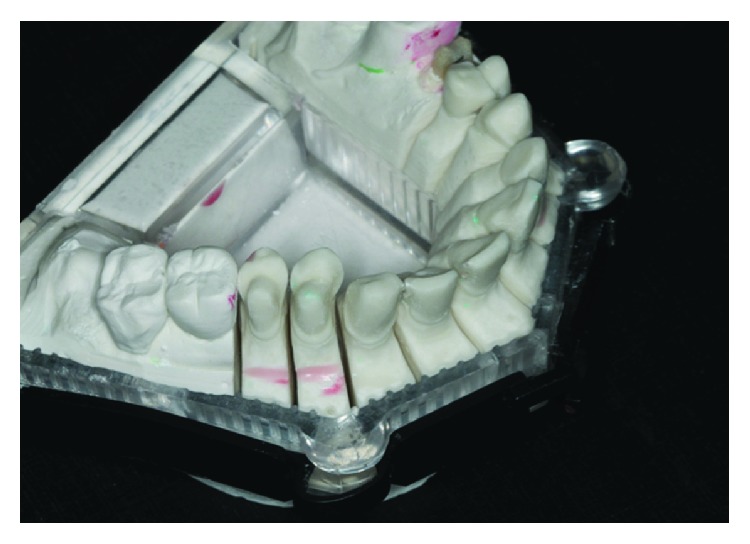
Die-cut model.

**Figure 8:**
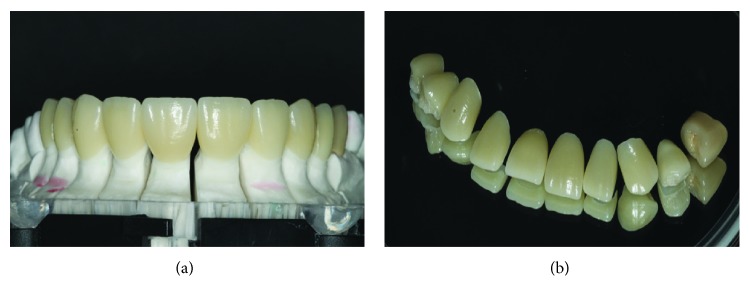
(a) Finished parts in the die-cut model. (b) Veneers in feldspathic ceramics.

The working model was obtained using stone rock type IV (Elite Rock, Zhermack) and then punched (Figure 7) through the Accutrac system (Coltene/Whaledent), in laboratory, for later duplication and confection of the veneers in feldspathic ceramics (IPS Empress II, Ivoclar Vivadent) (Figure 8). Once tested, adjusted, and approved, the cement agent color was chosen. In order to do so, temporaries were removed and the cement was tested using the try-in system (Variolink II, Ivoclar Vivadent), which are glycerin-based compounds that simulate the cement final color. Thus, based on this choice, the cement was finally selected. Once the cement color (color A3) was chosen, pieces were taken for cementation. Firstly, the try-in was removed, washing it under running water, and, subsequently, 10% hydrofluoric acid was applied (Ceramic Etching Gel, Ivoclar) for 60 seconds. The samples were then washed thoroughly in running water, and after a two-hour drying process, two different layers of silane (Monobond-Ivoclar) were applied for 60 seconds. After the piece preparation, isolation and gingival separation were done, using retractor wire # 000 (Ultrapak, Ultradent), embedded in hemostatic solution (aluminum chloride, Dentsply). The process was then accomplished on the buccal face of the teeth that would receive the veneer. The dental substrate was degreased, with detergent solution (Tergensol, Inodon), conditioned with 37% phosphoric acid (Condac, FGM) for 15 seconds, and then washed for 45 seconds for subsequent drying, leaving the substrate slightly moist. The adhesive system (Gluma, Heraeus Kulzer) was applied for 30 seconds, waiting for the adhesive to penetrate in the conditioned dentin tubules. A light air jet was applied, and then, each tooth was light cured for 30 seconds.

**Figure 9:**
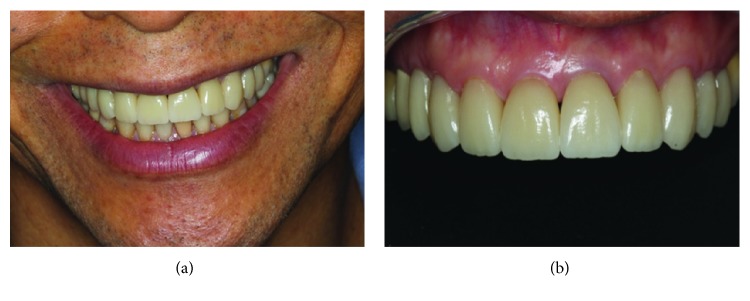
(a) Patient smile at the end of treatment. (b) Intraoral view of cemented parts.

The light-cured resin cement (Variolink II, Ivoclar Vivadent) was activated and placed inside the pieces that were placed into position and light cured again for additional two seconds. The excess cement was removed, using a scalpel blade, and the curing cycle was terminated (40 seconds on each side). Finally, a glycerin layer (Liquid Strip, Ivoclair Vivadent) was applied, on the cervical region, between the union piece and tooth, and resin cement was light cured for another 20 seconds, aiming at blocking the oxygen entrance. The excess cement was removed with the aid of scalpel blade number 15c (Solidor) and polished with silicone cups and felt disks (Figure 9).

After completing the case, the patient evaluated the final result of the installed work, comparing the before and after the installation of the ceramic veneers, and he was very satisfied with the result.

## 3. Discussion

Nowadays, there are many treatment options to restore aesthetically compromised anterior teeth. For many years, full-crown coverage was indicated for such situations; however, this process is considered very invasive due to the need to remove a large amount of healthy tissue [7]. Advances in the adhesive technologies have allowed the improvement of porcelains' shear bond strength in conditioned enamel/dentin, making it possible to use the conservative restoration techniques such as laminated veneers, which present ceramic color stability, biocompatibility, and good mechanical properties [3, 5, 7–12].

Technology and material advances have led to gradual changes in the indication of veneers, which are currently used for the following:

- Correction of alterations in the shape or position of teeth
- Teeth with enamel hypoplasia
- Closing diastema
- Fractured anterior teeth
- Previous guide rehabilitation
- Teeth with chromatic alteration
- Correction of intrinsic stains (such as stains caused by tetracycline) [1, 3, 5, 7, 9, 12, 13]

The conservative use of ceramic veneers provides satisfactory aesthetic results, preserving the structure of the teeth [8]. For this purpose, a carefully defined treatment plan and good communication between the dentist and the prosthetic lab help to maximize the success of treatment. According to Alhekeir et al. [12], insufficient clinical skills and/or operator inexperience often result in failures such as color change, cracking, fracture, and/or debonding [6, 11]. Models with diagnostic wax-up and mock-ups are used to define form and shape and assist to plan the case. Therefore, the patient previously sees the proposed final result, being able to request contour alterations to the clinician [5, 6, 9, 13]. The preparation of the teeth influences the hardness and color (translucence and shade) of the ceramic restoration, since the preparation determines the contour and the thickness of the ceramic material to be used. The desired position, color, and shape of the restoration should be the main determinants of the reduction level. This stage is established by the evaluation of the teeth condition versus the end result to be achieved [3, 5].

Although the recent results of dentin adhesive systems are promising, the bond strength between the porcelain and the enamel is higher when compared to the bond strength between the porcelain and the dentin [3].

There are several methods to obtain the necessary reduction as the free hand, which is the use of grooves and silicone guides that allow a visualization of the reduction needed to reach the shape and contours of the preplanned shape [8, 13]. Different models of tooth preparation have been described, differing only in the incisal third, overlap (palatine chamfer), and incisal reduction with the elimination of the palatine chamfer, resulting in stronger restorations and preparation of the simplified tooth [2, 3, 5]. Regarding the use of the best preparation, there is a disagreement among the authors, as some believe that the palatine chamfer is necessary to reinforce the ceramic veneer, since studies of the clinical behavior of this preparation showed results with greater resistance to fracture when compared to the preparations with 1 mm of incisal reduction with top joint or nonincisal preparation [12]. In the cervical third, the preparation should be located at the gingival level or, slightly, subgingival to the anterior teeth [3]. Sometimes, it may be preferable to extend the preparation beyond the points of contact, towards the surface of the palate, to hide the restoration margin [7]. The minimum thickness of a porcelain laminate veneer is 0.3 to 0.5 mm [13].

Gingival retraction, using retraction wires, is generally necessary for the preparation of the laminated veneers, as the cervical termination is located, a little below or, at the line, of the gingival margin [13].

The use of temporaries, after dental preparation, should be evaluated considering the extent of such preparation. Temporaries are not necessary if the patient agrees to remain without them [9, 13]. When there is dentin exposure, temporaries are made to protect the dental tissues against thermal injuries and chemical irritations, protecting against bacterial invasion and reestablishing tooth shape. For this purpose, acrylic resin and silicon mock-ups are used. Preparations can also be made with light-curing composite resins, with only one or two etching points on the buccal surface [4, 7, 8, 10, 13].

The ceramic properties, color stability, bond strength, clinical longevity, aesthetics, and biocompatibility with the periodontal tissues make this material a good choice of treatment [1, 3, 5, 8]. Feldspathic ceramics consist, predominantly, of silica powder or quartz, in a ratio of 46–66% aluminum oxide and liquid glass-based materials. These ceramics offer great aesthetic effect and high translucency, but the main issue is that they are fragile (low fracture resistance: 56.5 MPa), susceptible to fracture under mechanical stress. Nowadays, with less invasive treatments and higher levels of aesthetics, the use of feldspathic ceramics has been indicated for anterior teeth restorations, as a thickness of 0.5 mm is obtained. They are also indicated for cases with a substrate showing favorable coloring and for veneers that will not receive loads [1, 3].

Success in the cementing stage depends on the proper preparation, conditioning of the surfaces involved, the ceramic, the dental tissue, and the cementing agent. The dental surface (enamel and dentin) should be conditioned with 37% phosphoric acid and washed thoroughly. Correct drying is difficult to be obtained, as some moisture is required for the surface, being essential for the successful of the bonding process using dentin adhesives. At this stage, special care should be taken to avoid contamination with the saliva and the breathing moisture, which can reduce the enamel surface energy. Therefore, insulation with a rubber dam is highly recommended. The treatment of the ceramic surface differs according to its composition, but all surfaces should be conditioned with hydrofluoric acid, followed by silane application, differing just in the time of application. The silanization provides the chemical bond between the composite and the porcelain [3].

## 4. Conclusion

It can be concluded that using good planning using technology and following the sequence of clinical steps can achieve a clinical predictability with good functional and aesthetic result, preserving dental structures in the use of feldspathic ceramic veneers.
